# tRNA-derived RNA fragments in cancer: current status and future perspectives

**DOI:** 10.1186/s13045-020-00955-6

**Published:** 2020-09-04

**Authors:** Mengqian Yu, Bingjian Lu, Jisong Zhang, Jinwang Ding, Pengyuan Liu, Yan Lu

**Affiliations:** 1 Department of Respiratory Medicine, Sir Run Run Shaw Hospital and Institute of Translational Medicine, Zhejiang University School of Medicine, Zhejiang, 310029 Hangzhou China; 2 Center for Uterine Cancer Diagnosis & Therapy Research of Zhejiang Province, Women’s Reproductive Health Key Laboratory of Zhejiang Province, Department of Gynecologic Oncology, Women’s Hospital and Institute of Translational Medicine, Zhejiang University School of Medicine, Hangzhou, China; 3 Department of Head and Neck Surgery, Cancer Hospital of the University of Chinese Academy of Sciences, Zhejiang Cancer Hospital, Key Laboratory of Head & Neck Cancer Translational Research of Zhejiang Province, Hangzhou, China; 4 Center of Systems Molecular Medicine, Department of Physiology, Medical College of Wisconsin, Milwaukee, WI 53226 USA

**Keywords:** Biomarkers, Cancer, Epigenetic regulation, RNA silencing, Translation regulation, tRNA-derived fragments

## Abstract

Non-coding RNAs (ncRNAs) have been the focus of many studies over the last few decades, and their fundamental roles in human diseases have been well established. Transfer RNAs (tRNAs) are housekeeping ncRNAs that deliver amino acids to ribosomes during protein biosynthesis. tRNA fragments (tRFs) are a novel class of small ncRNAs produced through enzymatic cleavage of tRNAs and have been shown to play key regulatory roles similar to microRNAs. Development and application of high-throughput sequencing technologies has provided accumulating evidence of dysregulated tRFs in cancer. Aberrant expression of tRFs has been found to participate in cell proliferation, invasive metastasis, and progression in several human malignancies. These newly identified functional tRFs also have great potential as new biomarkers and therapeutic targets for cancer treatment. In this review, we focus on the major biological functions of tRFs including RNA silencing, translation regulation, and epigenetic regulation; summarize recent research on the roles of tRFs in different types of cancer; and discuss the potential of using tRFs as clinical biomarkers for cancer diagnosis and prognosis and as therapeutic targets for cancer treatment.

## Introduction

The intricate molecular mechanisms of tumorigenesis and development have always been a prime focus for cancer research. In addition to protein-coding messenger RNAs (mRNAs), research over the past few decades has elucidated roles for non-coding RNAs (ncRNAs), including long non-coding RNAs (lncRNAs) and small non-coding RNAs (sncRNAs), in various biological processes. ncRNAs’ involvement in complex mechanisms that play crucial roles in the development and progression of cancers [1–4] has been elucidated, thus challenging the previous views of these molecules as merely transcriptional “junk.”

In recent years, with the development of high-throughput sequencing technology and improvements in bioinformatics analysis, a new class of sncRNAs derived from tRNAs has been discovered. These tRNA-derived ncRNAs are called tRNA fragments (tRFs). Far from being random tRNA degradation products [5–7], the biogenesis of tRFs is actually controlled by a set of highly conservative and precise site-specific cutting mechanisms that produce transcripts that are 14–50 nucleotides in length [8–11].

There is increasing evidence that tRFs can regulate gene expression at transcriptional and post-transcriptional levels. tRFs participate in various molecular processes such as gene silencing, RNA processing, and protein translation and different physiological processes such as cell stress, cell growth, and cell differentiation [5, 12–15]. tRFs also play significant roles in various human diseases, including cancer [16, 17], neurodegenerative disease [18, 19], virus infection [20–22], metabolic disorder [23], and inflammation [24]. Here, we will review the biogenesis and discovery of tRFs and delineate some of the major biological functions of tRFs as well as recent reports on the roles of tRFs in different types of cancer. Finally, we will explore the potential of using tRFs as clinical biomarkers for cancer diagnosis and prognosis and therapeutic targets for cancer treatment.

## Biogenesis and discovery of tRFs

**Fig. 1:**
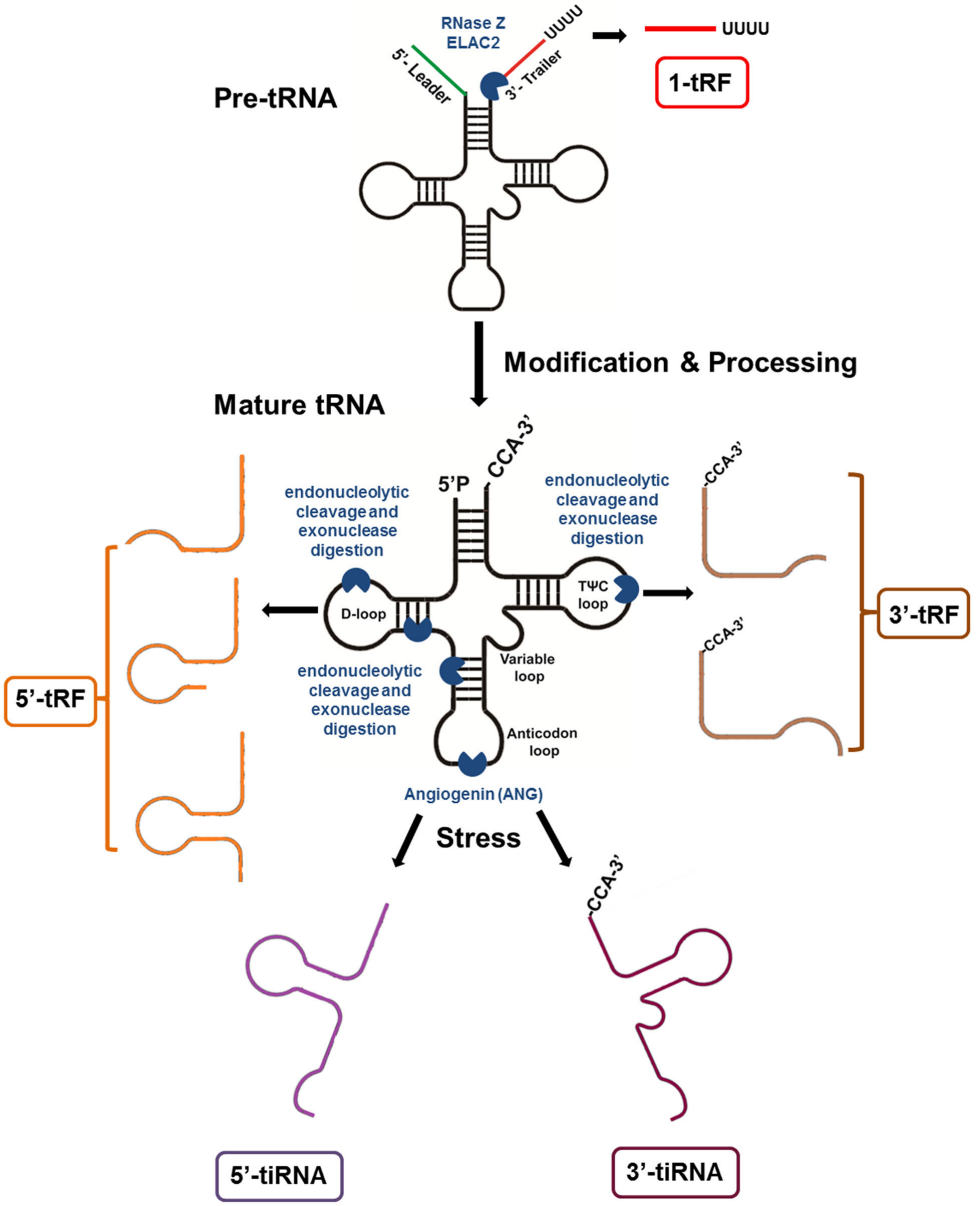
Different types of tRNA-derived RNA fragments produced from either pre-tRNAs or mature tRNAs. The 1-tRF series is produced by RNase Z (or ELAC2) cleavage of the pre-tRNA during the tRNA processing. Mature tRNA can be cleaved in the anticodon loop by ANG to produce 5′-tiRNA and 3′-tiRNA series under stress conditions. The 5′-tRF series is derived from the 5′-end of mature tRNAs by endonucleolytic cleavage and exonuclease digestion in the D-loop. The cleavage in the T-loop results in the production of the 3′-tRF series

Generally, tRFs are defined and named according to the cleavage positions on pre- and mature tRNAs in various cell types and organisms. These tRFs can be roughly classified into four categories (Fig. 1). tRNAs undergo extensive processing and a series of chemical modifications in their life cycle. During tRNA maturation, the 3′-trailer sequences are removed from pre-tRNA by the endonuclease Z (RNase Z, ELAC2), which results in the production of 1-tRF [8, 25]. The other two classes of tRFs are generated from mature tRNAs: 5′-tRF as produced by cleavage of the 5′ end in the D-loop and 3′-tRF as produced through cleavage of the 3′ end in the T-loop. The final longer tRF type, 30–50 bases in length, are called tRNA-derived stress-induced RNAs (tiRNAs) or tRNA halves and are generated by specific cleavage in the anticodon loops of mature tRNAs by angiogenin (ANG) under stress conditions, such as hypoxia, starvation, virus infection, arsenite, heat shock, or heavy metal-induced cellular stress/toxicity [15, 26–30]. As a stress-activated and secreted ribonuclease, ANG can be transported to the cytoplasm under stress conditions. In the cytoplasm, it cleaves tRNAs to produce tiRNAs, which is highly related to tRNA modifications [30, 31].

In recent years, the most commonly used techniques for identifying tRFs are deep sequencing [32, 33] and microarrays [34, 35]. Such large-scale discovery of tRFs has promoted the development of several tRF-related databases (Table 1). The first, tRFdb, was the first attempt to present the tRF sequences and read counts from eight species, including humans, and is available at http://genome.bioch.virginia.edu/trfdb/ [7]. Then came MINTbase, which is a repository tabulating tRF information that arises from the nucleic and mitochondrial tRNAs and is freely accessible at http://cm.jefferson.edu/MINTbase/. It contains information about sequence, expression abundance, parental tRNAs, and other genomic information [36]. Another study identified hundreds of distinct tRFs in the National Cancer Institute 60 (NCI-60) cell lines and TCGA tumor samples. The expression profile of these tRFs was compiled into a public database, tRFexplorer (https://trfexplorer.cloud/) [37]. Another web server, named tRF2Cancer (http://rna.sysu.edu.cn/tRFfinder/), can be used for identifying tRFs from small RNA sequencing datasets from various cancer types [10]. However, these databases only contain tRF expression across cancer types or focus on the identification of tRFs from small RNA sequencing data. We recently integrated large-scale small RNA sequencing, RNA sequencing, clinicopathologic datasets from TCGA, information from chemical modification sites on parental tRNAs, and from other validated literature manually curated from PubMed, and used these to construct a comprehensive database named OncotRF (http://bioinformatics.zju.edu.cn/OncotRF). OncotRF adopts a highly conserved filtering strategy in which only the tRFs with 10th quantile reads per million (RPM) > 1 are retained in the reported candidate list. This ensures robust results in downstream analysis. It provides a valuable tRF resource for users to identify diagnostic and prognostic biomarkers, develop cancer therapy, and study cancer pathogenesis [38].

**Table 1:** tRF databases

Database	Description	URL link
tRFdb [7]	Present the tRF sequences and read counts from eight species, including humans.	http://genome.bioch.virginia.edu/trfdb/
MINTbase [36]	A repository tabulating tRF information that arises from the nucleic and mitochondrial tRNAs.	http://cm.jefferson.edu/MINTbase/
tRFexplorer [37]	Show the expression profile of tRFs in every cell line in NCI-60 as well as for each TCGA tumor type.	https://trfexplorer.cloud/
tRF2Cancer [10]	Identify tRFs from small RNA sequencing datasets from various cancer types.	http://rna.sysu.edu.cn/tRFfinder/
OncotRF [38]	Provide the most comprehensive tRF resource relating to human cancers including exploration of tRF function and identification of diagnostic and prognostic biomarkers.	http://bioinformatics.zju.edu.cn/OncotRF

Although standard deep sequencing methods are used to discover tRFs and quantify their abundance, it has been reported that tRNA (and tRF) has been heavily modified, and these chemical modifications may affect the detection and quantification of tRFs using standard sequencing methods [33, 39]. Several newly developed methods may overcome this obstacle such as AlkB-facilitated RNA methylation sequencing (ARM-seq) [40] and engineered demethylases-based tRNA sequencing (DM-tRNA-seq) [41]. Besides the classical northern blot assay, qRT-PCR was used to quantitatively analyze the abundance of individual tRFs after removing the tRNA modifications [23, 42]. We recently improved dumbbell-PCR (Db-PCR) to detect tRFs, which is a TaqMan qRT-PCR-based method and can distinctively quantify 5′ and 3′ end variants of RNA fragments at the single base resolution [43] (unpublished). In addition, most current studies usually ignore the important role of tRNA modifications when using synthetic RNAs to mimic endogenous tRFs. A recent study developed a simple method to isolate tiRNAs in vivo, which may facilitate functional studies of tiRNAs by addressing this modification problem [30].

## Biological functions of tRFs

**Fig. 2:**
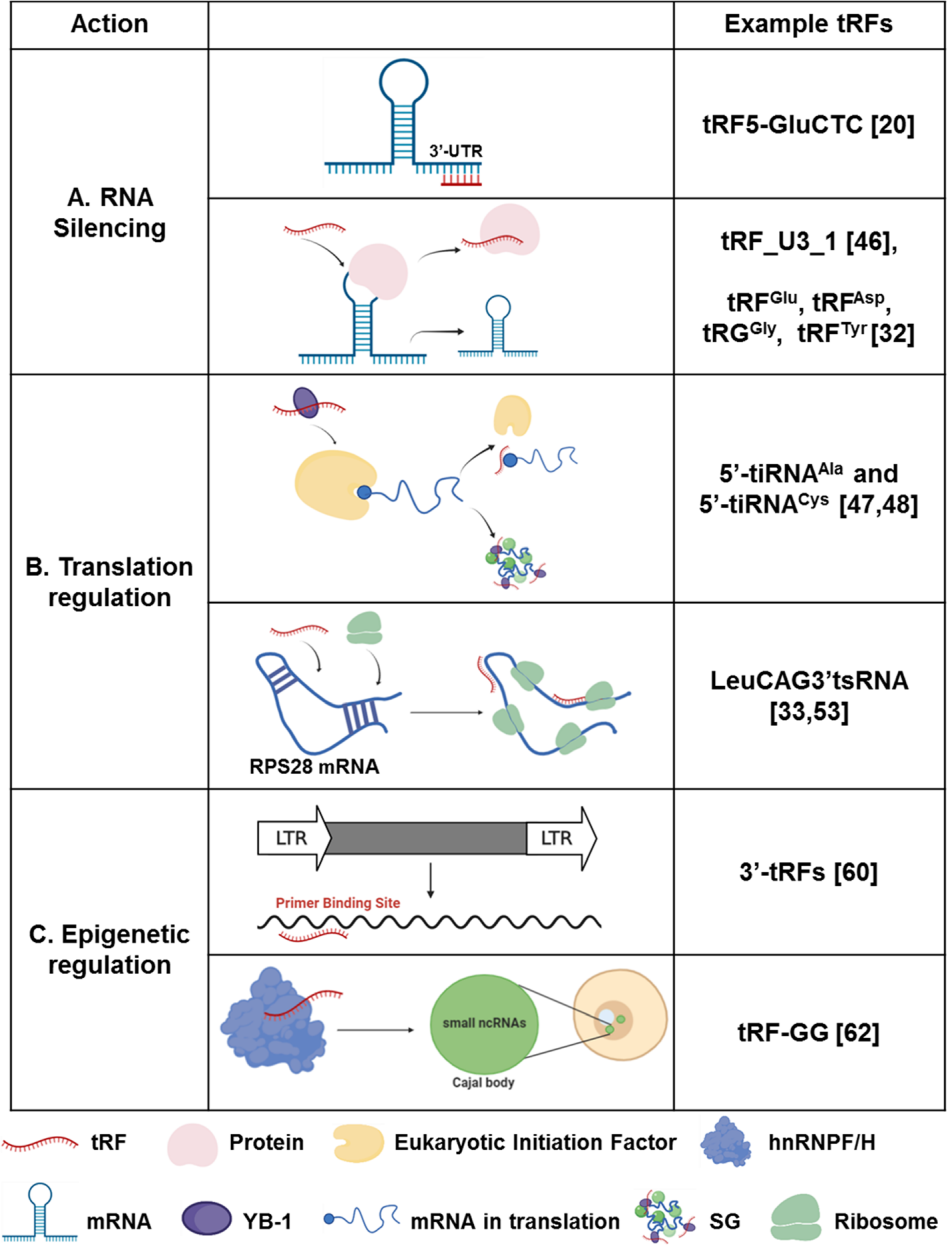
Biological functions of tRFs. (A) RNA splicing. tRFs can affect RNA splicing by targeting the 3′-UTR regions of mRNAs or competitive binding of target mRNAs. (B) Translation regulation. YB-1 binding tRFs repress global translation by displacing translation eukaryotic initiation factor and induce assembly of SGs. tRFs can also regulate translation by interacting with ribosomes. (C) Epigenetic regulation. tRFs can inhibit LTR-retrotransposons or participate in non-coding RNA regulation

Though the biological functions of tRFs are complex and require further elucidation, our current knowledge of their function, as presented here, has been summarized into three categories: RNA silencing, translation regulation, and epigenetic regulation (Fig. 2). These three categories of tRF function have also been a key focus in cancer research.

### RNA silencing

Analysis of a human Photoactivatable-Ribonucleoside-Enhanced Cross*l*inking and Immuno*p*recipitation (PAR-CLIP) data has revealed that some 5′-tRFs and 3′-tRFs bind to Argonautes (AGO) in a manner similar to miRNAs, with the exception that they preferentially bind to AGO1, 3, and 4 rather than AGO2. Most 5′-tRFs and 3′-tRFs have been shown to interact with RNAs in the cells, suggesting that the majority of tRFs may play important roles in RNA interference (RNAi)-mediated silencing [9]. The observation that the regulation of tRF level has significant effects on the silencing activities of microRNAs (miRNAs) and small interfering RNAs (siRNAs), rather than on the abundance of miRNAs and siRNAs, also suggested tRF involvement in the global control of small RNA silencing [44]. In addition, 3′-tRFs are seen to post-transcriptionally repress genes in HEK293T cells. This 3′-tRF-mediated repression is Dicer-independent, but AGO-dependent, and targets are recognized by sequence complementarity [45]. All of these studies suggest that tRFs play a role in gene silencing by directly targeting mRNAs in a manner similar to miRNAs, or even competing with miRNAs to bind to their targets. Considering the difference between tRFs and miRNAs, a study has developed an unbiased approach that combines biochemical screening, analysis of gene expression data, and in silico prediction to identify tRF targets. It was found for the first time that the endogenous target of a 5′-tRF (derived from tRNA-Glu-CTC) can promote respiratory syncytial virus replication by targeting the 3′-UTR region of the APOER2 gene [20].

Another mechanism for gene silencing by tRFs is through competitive binding of target proteins with mRNAs. For example, unlike the above mechanism of tRF in viral infections, a chr10.tRNA2-Ser(TGA)-derived 1-tRF, tRF_U3_1, can bind directly to La/SSB protein and inhibit La/SSB-dependent viral gene expression [46]. This means that tRFs may have involvement in multiple different mechanisms within the same disease.

### Translation regulation

Although tRNAs are essential components of translational machinery, the mode of translation regulation by tRFs is not simply the result of changes in the amount of mature tRNAs involved in the synthesis of proteins.

Stress-induced 5′-tiRNAs, but not 3′-tiRNAs, were found to inhibit protein synthesis [26] and trigger the phospho-elF2α-independent assembly of stress granules (SGs) [14]. Further investigations clarified that 5′-tiRNAs, such as 5′-tiRNA^Ala^ and 5′-tiRNA^Cys^, can repress global translation by displacing translation eukaryotic initiation factor eIF4G and eIF4A from mRNAs and displacing eIF4F from isolated m^7^G cap [47]. This 5′-tiRNA-associated translational silencer YB-1 contributes to the induction of SG assembly and stress-induced translational repression [47]. However, YB-1 was later noted to directly bind to tiRNAs by its cold shock domain, which is indispensable in packaging tiRNA-repressed mRNAs into SGs, but is not necessary for tiRNA-mediated translational inhibition [48].

A further important breakthrough came when Ivanov et al. showed in 2014 that translationally active 5′-tiRNAs assembled unique G-quadruplex structures that are essential for translational suppression [49]. Lyons’ group later added the observation that RNA G-quadruplex (RG4), a critical component of cellular stress response, is found to be required for functions of tRFs in the regulation of mRNA translation, and where the destruction of RG4 deprives tRFs of the ability to trigger the formation of SGs in vivo [50]. More recently still, Gkatza et al. showed that the loss of NSUN2, a cytosine-5 RNA methyltransferase, altered the biogenesis of tiRNAs in response to stress, leading to impaired regulation of protein synthesis [51].

In addition to tiRNAs, a universal conserved “GG” dinucleotide in 5′-tRFs is noted to also inhibit the process of protein translation [52]. tRFs can also regulate translation by interacting with ribosomes. One specific 3′-tRF derived from Leu-CAG tRNA (LeuCAG3’tsRNA) was seen to bind to a coding and non-coding 3′-UTR sequence in the ribosomal protein S28 (*RPS28*) mRNA to enhance its translation and ultimately the number of ribosomes. This may represent a conserved tRF-regulated translational mechanism among vertebrates [33, 53]. This ribosome-associated translation regulation mechanism is also observed in other invertebrate organisms, including the *Trypanosoma brucei* parasite [54] and the archaeon *Haloferax volcanii* [55].

Alternatively, epigenetic modifications can affect tRF’s role in translation control. For example, PUS7, the “writer” of pseudouridylation, can modify and activate a novel network of tRF targeting the translation initiation complex. Inactivation of PUS7 in embryonic stem cells weakens tRF-mediated translation regulation, leading to an increase of protein biosynthesis and the defect of germ layer specification [56].

### Epigenetic regulation

The expression of biogenetic information is controlled by both DNA sequence and epigenetic information. Epigenetics regulate gene expression mainly through DNA methylation, histone modification, chromatin remodeling, and ncRNA regulation [57, 58]. Several studies have demonstrated that tRFs can regulate gene expression by affecting different epigenetic processes.

Transposable elements (TEs) and their repetitive sequences contribute to the formation and function of chromosomes, induce epigenetic regulation of specific genes, and drive transcription. However, TE mobility is driven by intact, active transposons and is highly mutagenic, necessitating tight control [59]. Transcription of TEs is generally repressed by epigenetic marks such as histone modification and DNA methylation. In the absence of epigenetic transcriptional suppression, 3′-tRFs can strongly inhibit long terminal repeat (LTR)-retrotransposon or endogenous retrovirus (ERV) activity in mice by targeting the highly conserved primer binding site of the LTR-retrotransposons [60].

tRFs can also participate in ncRNA regulation. tRNA methyltransferase Dnmt2 limits the extent of tRNA fragmentation in heat shock responses. The resulting tRFs can inhibit the activity of Dicer-2 on long double-stranded RNAs (dsRNAs). Consequently, heat-shocked Dnmt2 mutations lead to the accumulation of dsRNAs and the production of fewer siRNAs, resulting in dysregulation of siRNA pathway-dependent genes [61]. Recently, Boskovic et al. identified a specific 5′-tRF, tRF-GG, that plays a role in the production of a wide variety of small ncRNAs, the stability and activity of which were seen as dependent on Cajal bodies by directly binding to RNA-binding proteins hnRNPF/H. Importantly, the regulation of the U7 snRNA by tRF-GG regulated the heterochromatin-mediated transcriptional repression of endogenous retroelement MERVL elements by providing sufficient histone proteins [62].

## Roles of tRFs in cancer

**Fig. 3:**
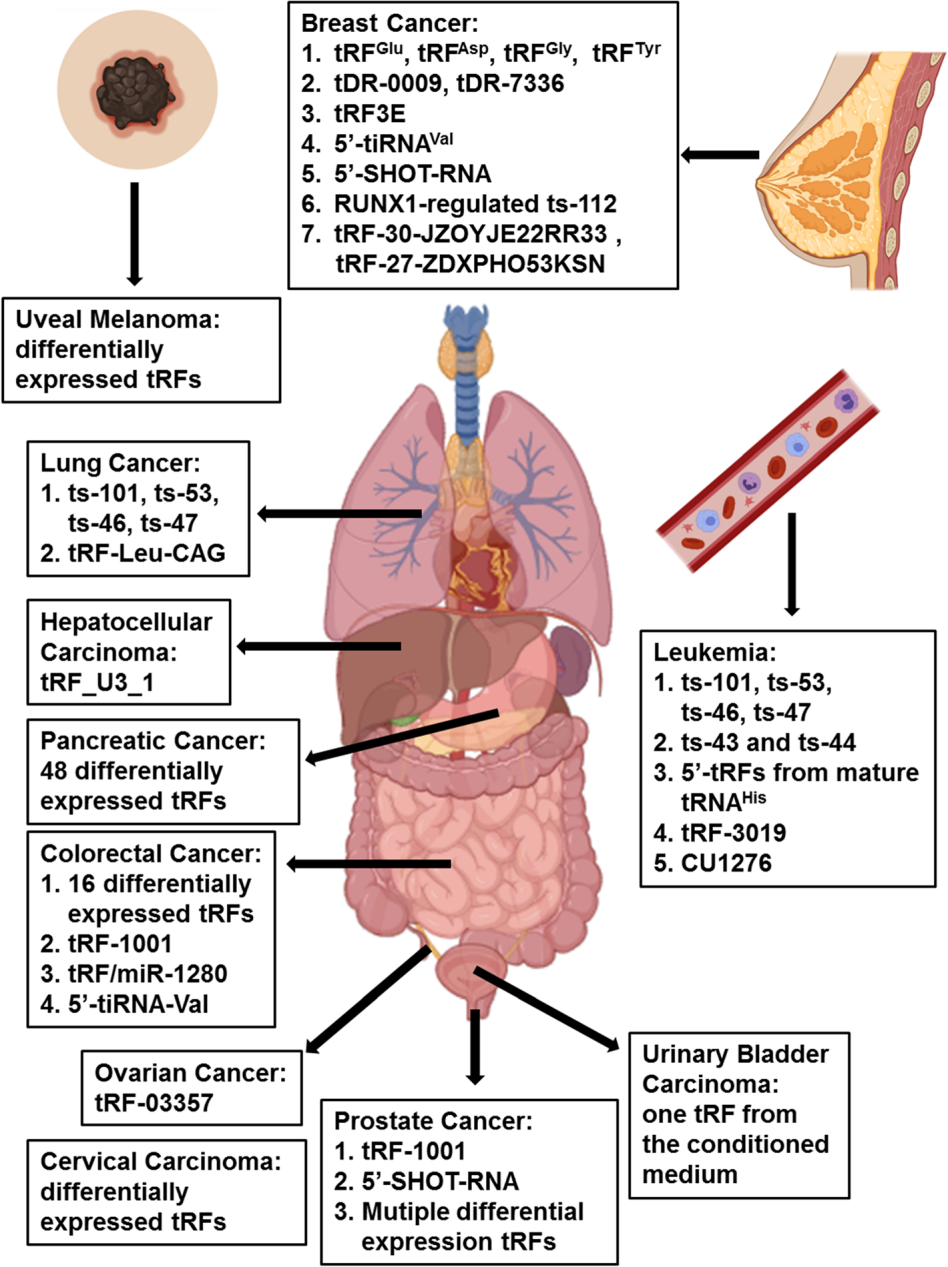
Roles of tRFs in different types of cancer. tRFs are associated with many types of cancer including breast cancer, prostate cancer, leukemia, lung cancer, colorectal cancer, hepatocellular carcinoma, ovarian cancer, urinary bladder carcinoma, cervical carcinoma, uveal melanoma, and pancreatic cancer. These tRFs can play differing biological functions in different types of cancer

Growing evidence indicates that tRFs contribute to various biological processes associated with cancer development and progression. We will now proceed to describe recent advances in the study of biological functions and the underlying molecular mechanisms of dysregulated tRFs and the associated machinery in the pathogenesis of various types of cancers (Fig. 3 and Table 2).

**Table 2:** Functional tRFs in different types of cancer

Cancer type	tRF name	Role	Function	Ref
Breast cancer	tRF^Glu^, tRF^Asp^, tRF^Gly^, tRF^Tyr^	Tumor suppressor	Destabilization of pro-oncogenic transcripts via YBX1 displacement.	[32]
	tDR-0009, tDR-7336	Upregulate under hypoxia conditions	Facilitate the doxorubicin resistance in TNBC cells.	[63]
	tRF3E	Tumor suppressor	Promote p53 translation through competitive interactions with nucleolin.	[64]
	5′-tiRNA^Val^	Tumor suppressor	Inhibit the *FZD3*-mediated Wnt/β-Catenin signaling pathway in BC cells.	[65]
	5′-SHOT-RNA	Oncogene	Enhance cell proliferation in BC cells.	[66]
	ts-112	Oncogene	Promote cell growth in normal-like mammary epithelial cells.	[67]
	tRF-30-JZOYJE22RR33, tRF-27-ZDXPHO53KSN	Upregulate in trastuzumab-resistant patients	Correlate with trastuzumab resistance in HER-2-positive BC.	[42]
Prostate cancer	tRF-1001	Oncogene	Promote the proliferation of prostate cancer cells.	[68]
	5′-SHOT-RNA	Oncogene	Promote the proliferation of prostate cancer cells.	[66]
Leukemia	ts-101, ts-53, ts-46, ts-47	Downregulate in CLL samples	Interact with Ago proteins and Piwil2.	[35]
	ts-43, ts-44	Downregulate in CLL samples	Likely act as a tumor suppressor.	[69]
	tRF-3019	Detected in HTLV-1 infection cells	Match the sequence of the primer binding site of HTLV-1 and activate HTLV-1 reverse transcriptase.	[70]
	CU1276	Upregulate in normal germinal center B cells	Inhibit endogenous RPA1 to inhibit cell proliferation and regulate the molecular response to DNA damage.	[71]
Lung cancer	ts-101, ts-53, ts-46, ts-47	Tumor suppressor	Inhibit the colony formation of lung cancer cells.	[35]
	tRF-Leu-CAG	Oncogene	Repress AURKA to promote cell proliferation and cell cycle progression.	[72]
Colorectal cancer	tRF-1001	Oncogene	Promote the proliferation of HCT-116 cells.	[68]
	tRF/miR-1280	Tumor suppressor	Interact with the 3′-UTR of Notch ligand JAG2 to repress Notch/Gata and miR-200b signaling and inhibit CRC growth and metastasis.	[73]
	5′-tiRNA-Val	Highly expressed in CRC patients	Regulate ANG-mediated CRC metastasis.	[74]
Hepatocellular carcinomas	tRF_U3_1	Upregulate in the HCC cell line and tissues	Inhibit viral gene expression and its precursors.	[46]
Ovarian cancer	tRF-03357	Oncogene	Promote the cell proliferation, migration, and invasion of high-grade serous ovarian cancer partly by downregulating HMBOX1.	[75]
Urinary bladder carcinomas	One tRF	Isolated from conditioned medium of human urinary bladder carcinoma cells	Inhibit the growth of endothelial cells.	[76]

### Breast cancer

**Fig. 4:**
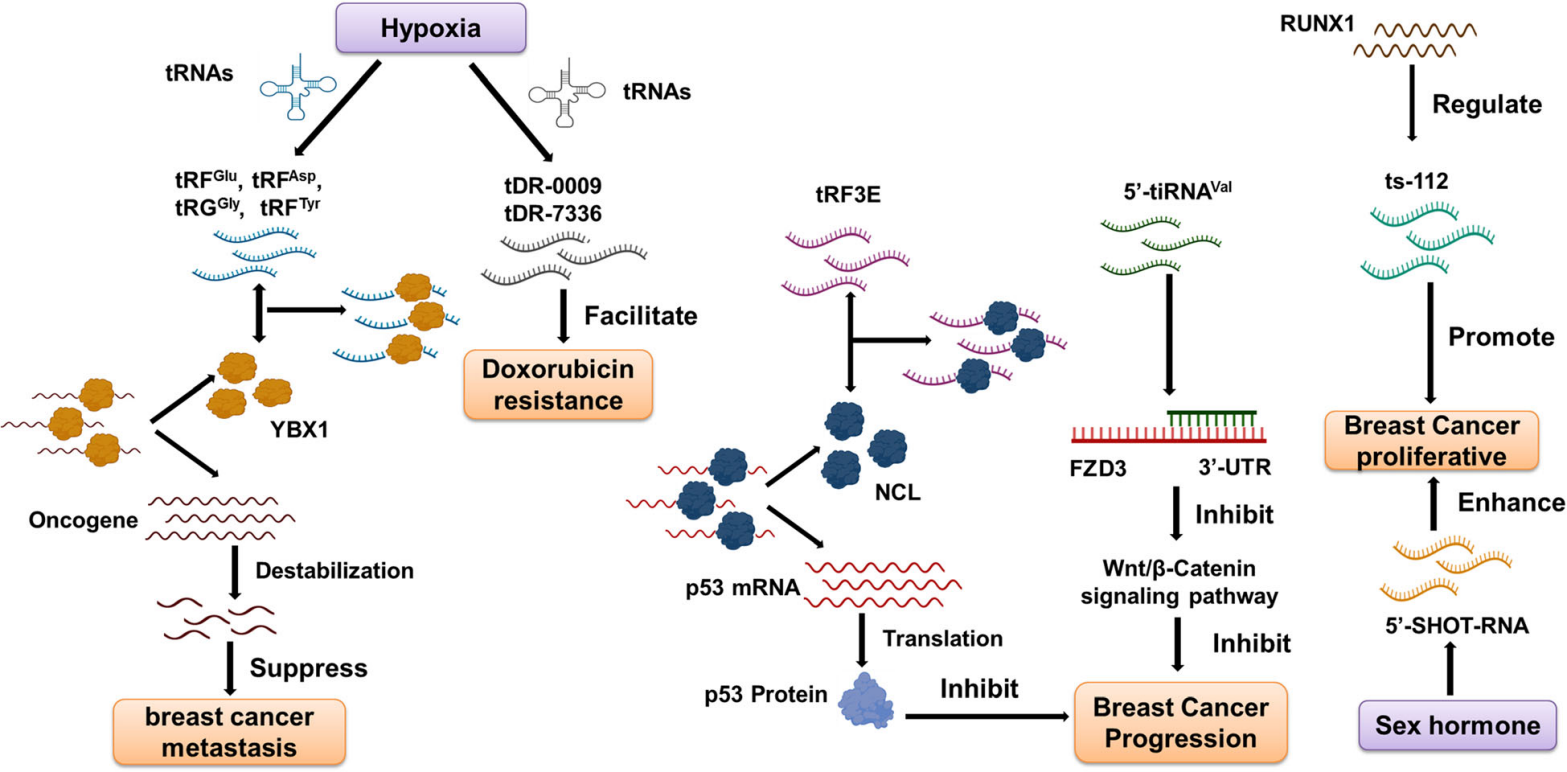
Mechanisms of action of tRFs in breast cancer. Hypoxia-induced tRNA^Glu^, tRNA^Asp^, tRNA^Gly^, and tRNA^Tyr^ can interact with YBX1 and suppress breast cancer metastasis. Hypoxia-induced tDR-0009 and tDR-7336 can facilitate the doxorubicin resistance in triple-negative breast cancer cells. tRF3E can inhibit cell proliferation by binding with NCL. 5′-tiRNA^Val^ inhibits breast cancer progression by directly targeting FZD3 3′-UTR sequence. RUNX1-regulated tRFs and sex hormone-dependent tiRNA (SHOT-RNAs) are associated with cell proliferation

A few tRFs have been noted as differentially expressed in breast cancers (BC) [35]. Many mRNAs are also differentially expressed between normal breast and triple-negative breast cancer (TNBC) in tandem with isomiR or tRF dysregulation [77]. All of these implicate tRFs as potential candidates for BC diagnostic and prognostic biomarkers and therapeutic targets. The role of tRFs in BC has received increasing attention over the past few years (Fig. 4).

Hypoxic stress induces the production of a new class of tRFs derived from tRNA^Glu^, tRNA^Asp^, tRNA^Gly^, and tRNA^Tyr^. This class of tRFs can suppress the development of BC metastasis by binding to the oncogenic RNA-binding protein YBX1 and displacing multiple oncogenic transcripts such as EIF4EBP1 and AKT1. This results in the suppression of their stability [32]. tDR-0009 and tDR-7336, which were significantly upregulated under hypoxia conditions, have been found to facilitate the doxorubicin resistance in TNBC cells [63]. These data indicate that hypoxia-induced specific tRFs may act either as tumor suppressors or as a novel class of regulatory factors involved in hypoxia-induced chemoresistance.

Other tRFs that act as tumor suppressors have also been identified. tRF3E, a 3′-tRF derived from tRNA-GluTTC, is expressed in healthy mammary glands but not in BC. The level of serum tRF3E in patients with HER2-positive BC decreased with the increase of malignancy. As a tumor suppressor, tRF3E causes the release of p53 mRNA and further promotes p53 translation through competitive interactions with nucleolin (NCL), an RNA-binding protein overexpressed in BC, resulting in inhibition of cancer cell proliferation [64]. Another tRF, 5′-tiRNA^Val^, can inhibit the frizzled class receptor 3 (*FZD3*)-mediated Wnt/β-Catenin signaling pathway in BC cells by directly targeting the *FZD3* 3′-UTR sequence [65]. Its downregulation in serum was positively correlated with BC stage progression and lymph node metastasis.

Conversely, some tRFs have been found to act as oncogenes in BC. Sex hormone-dependent tRNA-derived RNAs (SHOT-RNAs) are specifically and abundantly expressed in ER-positive BC cell lines and patients. Similarly, 5′-SHOT-RNA can also enhance cell proliferation in BC cells [66]. Inhibition of ts-112, the expression of which is regulated by the tumor suppressor runt-related transcription factor 1 (RUNX1), can reduce the proliferative capacity of aggressive breast cancer cells while its overexpression promotes cell growth in normal-like mammary epithelial cells [67].

### Prostate cancer

Small RNA sequencing of prostate cancer revealed a pronounced increase in tRFs in prostate cancers that occurred with metastatic lymph nodes compared with more organ-confined disease [78]. Analysis of a tRF profile in a large cohort of prostate adenocarcinoma (PRAD) patients from The Cancer Genome Atlas (TCGA) also found that tRFs have extensive correlations with mRNAs which were disrupted in PRAD. Interestingly, the profile of tRFs differed in patients of different races [79].

These findings provide clues for work on the elucidation of any putative roles of tRFs in PRAD carcinogenesis. Such a work has begun where, for example, one report details a specific 1-tRF called tRF-1001 which can act as an oncogene in PRAD. tRF-1001 is present in the cytoplasm and is produced by tRNA 3′-endonuclease ELAC2 (a prostate cancer susceptibility gene), and it is required for the proliferation of prostate cancer cells [68]. In addition, the expression of 5′-SHOT-RNA (as previously mentioned) is also seen as promoted by sex hormones and their receptors in androgen receptor (AR)-positive prostate cancer cell lines and has significant functional involvement in cell proliferation [66].

### Leukemia

Studies have also begun to uncover tRF signatures in chronic lymphocytic leukemia (CLL). One such study demonstrated that ts-101, ts-53, ts-46, and ts-47 were all downregulated in CLL samples. ts-101 and ts-53 interact not only with Ago proteins but also with Piwil2, a protein involved in the silencing of transposons [35]. Similarly, another report revealed that ts-43 and ts-44, derived from distinct genes of pre-tRNAHis, as well as 5′-tRFs from mature tRNAHis, are all downregulated in CLL samples. Further investigations of the expression of tRFs in both aggressive CLL and indolent CLL revealed drastic dysregulation of the expression of mature tRFs in CLL [69]. These results suggest that tRFs are dysregulated and may have cancer-associated functions in CLL.

Several functional tRFs were also observed in other types of leukemia. For example, tRF-3019 perfectly matches the sequence of the primer binding site of human T cell leukemia virus type 1 (HTLV-1), the causative agent of adult T cell leukemia/lymphoma (ATLL). An in vitro reverse transcriptase assay further verified that tRF-3019 was capable of activating HTLV-1 reverse transcriptase and therefore may represent a new target to control HTLV-1 infection [70]. Another tRF, CU1276, was found abundant in normal germinal center B cells but absent in germinal center-derived lymphomas. Furthermore, the expression of CU1276 in a lymphoma cell line could inhibit cell proliferation and regulate the molecular response to DNA damage due to inhibition of endogenous replication protein A1 (RPA1), which is involved in many important aspects of DNA dynamics [71].

### Lung cancer

At present, research on the role of tRFs in lung cancer is still lacking, especially related to their molecular mechanisms. However, the four tRFs of the tRF signatures in CLL mentioned above (ts-101, ts-53, ts-46, ts-47) are also seen to be downregulated in lung cancer. Overexpression of ts-46 and ts-47 in two lung cancer cell lines strongly inhibited the colony formation of cells, confirming that tRFs can affect lung cancer cell growth and survival [35].

The level of tRF-Leu-CAG was higher in human non-small cell lung cancer (NSCLC) tumor tissues than in normal tissues. Inhibition of tRF-Leu-CAG in H1299 cells suppressed cell proliferation and impeded the cell cycle, possibly due to the repression of aurora kinase A (AURKA). AURKA can be also directly targeted by miR-137 and miR-32 to affect the progression of NSCLC [72]. However, whether AURKA is a direct target of tRF-Leu-CAG and whether tRF-Leu-CAG can interact with miR-137 or miR-32 still require investigation.

### Colorectal cancer

tRF-1001, as mentioned above, was also strongly associated with colon cancer cell proliferation. In the HCT-116 cell line, knockdown of tRF-1001 increased the proportion of cells in the G2 phase of the cell cycle and led to a significant decrease in cell viability [68]. A comprehensive small RNA sequencing study then identified 16 differentially expressed tRFs between colon cancer and paired paracancerous tissue. Meanwhile, 55 differentially expressed mRNAs were identified as potential targets of these tRFs and were noted as primarily enriched in vitamin metabolic pathways and the cyclic guanine monophosphate-protein kinase G signaling pathway [80].

Relating to the metastasis of colorectal cancer (CRC), one study demonstrated that tRF/miR-1280, derived from both tRNA-Leu and pre-miRNA, suppresses Notch/Gata and miR-200b signaling through a direct interaction with the 3′-UTR of Notch ligand JAG2 and plays important roles in CRC growth and metastasis [73]. Another study revealed that increased ANG to CRC growth and metastasis was due to the production of ANG-cleaved tiRNAs. Among them, a 5′-tiRNA from tRNA-Val was highly expressed in CRC patients and was closely related to tumor metastasis [74]. These results provide valuable cues for further insights into the roles of tRFs in colon cancer.

### Other cancers

In addition to the abovementioned cancers, functional tRFs have been associated with several other types of cancer, but the amount and depth of these studies are rather limited and further research is needed.

In hepatocellular carcinomas (HCC), tRF_U3_1 is more abundant in the HCC cell line Huh7 and cancerous liver tissues compared to primary hepatocytes and normal liver tissues, which can inhibit viral gene expression and its precursors [46]. Whether the inhibition of viral gene expression by tRF_U3_1 has an impact on HCC still requires verification.

For ovarian cancer, it has been suggested that tRF-03357 promotes cell proliferation, migration, and invasion of high-grade serous ovarian cancer partly by downregulating HMBOX1 [75]. This also requires confirmation.

For urinary bladder carcinomas, one study isolated a tRF from the conditioned medium of human urinary bladder carcinoma cells, which can inhibit the growth of endothelial cells, demonstrating a new in vitro role for tRFs as a selective endothelial cell inhibitor [76].

A direct sequencing method was also used to characterize miRNA profiles and other small RNA species from six human cervical carcinoma cell lines and five normal cervical samples. A significant expression variation in the number of tRFs was observed among these sequencing samples [81]. Furthermore, by comprehensively characterizing the small RNA profiles in 80 primary uveal melanoma tumor samples, researchers showed that the abundance profiles of isomiRs and tRFs were correlated with various molecular phenotypes, metastatic disease, and patient survival [82]. Coincidentally, another tRF sequencing study screened out a total of 48 tRFs in clinical pancreatic cancer samples [83]. All of these tRF profiling studies suggest that further investigation and characterization of these differentially expressed tRFs will improve our understanding of the mechanisms underlying these cancers.

## Potential clinical applications of tRFs

Although tRF researches are still in their infancy, tRFs have gradually become clinical biomarkers for cancer diagnosis and prognosis and therapeutic targets for cancer treatment. In this section, we will discuss the potential of tRFs in clinical application.

### Clinical biomarkers for cancer diagnosis and prognosis

Early detection, diagnosis, and treatment are always considered a key towards an improved prognosis for most cancers. At present, clinical and pathological methods for predicting postoperative clinical results remain limited. Therefore, it is essential to find accurate biomarkers for the early diagnosis and accurate prognosis of cancer. Recently, extracellular RNAs are gaining more and more clinical attention as non-invasive biofluid-based markers for diseases, especially cancer. In particular, tRFs are emerging as such biomarkers with high potentials.

As early as 1979, scientists discovered that tRNA breakdown products could be used as cancer biomarkers. They found 7 tRNA breakdown products in the urine of 26 patients with 13 different malignancies. The level of the excretion of these factors varied with the stage of the disease [84]. Nevertheless, it was not until recent years, with the development of high-throughput sequencing technology, that the potential of tRFs as biomarkers has been further explored. Several studies have demonstrated that tRFs can be present in a rich complex in serum [85], or are highly abundant in human saliva [86]. In addition, tRFs can also be selectively exported into extracellular vesicles (EVs) [87]. Some 5′-tRFs and tiRNAs have been found in exosomes and are upregulated during stress responses and cancer [88], suggesting that tRFs can circulate in the blood in a stable form and can act as novel forms of signaling molecules. For example, one study identified the high abundance of tRFs in EVs derived uniquely from the MCF7 breast cancer cell line [89].

Increasing numbers of examples of abnormal tRF expression are being discovered in various cancers. They are easy to detect in bodily fluids, and some are clear indicators of important biological functions in cancers. For example, as mentioned above, the expression of tRF3E [64] and 5′-tiRNA^Val^ [65] in the blood is related to the degree of malignancy of BC and may serve as diagnostic biomarkers. In particular, 5′-tiRNA^Val^ can be used to distinguish differentiated BC from healthy controls, with a sensitivity of 90.0% and a specificity of 62.7% [65]. Meanwhile, the detection of the functional tRF-Leu-CAG in the serum of NSCLC is related to progression stage, suggesting that it may be a new diagnostic marker and potential therapeutic target for NSCLC [72]. Circulating tRF-Lys and another two miRNAs in EVs can discriminate early-stage BCs at stage 0 from controls with an AUC (area under the curve, a criterion for judging the pros and cons of a binary classification prediction model) of 0.92 [90]. All these studies indicated that tRFs have great potential as clinical biomarkers for cancer diagnosis and prognosis.

### Therapeutic targets

As small molecules, certain tRF mimetics or antisense molecules against tRFs can be used as small molecule drugs [91], which makes certain tRFs also have great potential as therapeutic targets. For example, transfection of mimetics of four tRFs that bind YBX1 can significantly inhibit lung metastasis in vivo [32], indicating that mimetics of certain tRFs can be used to treat tumor progression and metastasis.

Furthermore, drug resistance has always been one of the main causes of cancer treatment failure. The causes of drug resistance are usually multifactorial, and there are still many challenges in improving the outcome of patients [92, 93]. In addition to the abovementioned tDR-0009 and tDR-7336 related to doxorubicin resistance [63], tRF-30-JZOYJE22RR33 and tRF-27-ZDXPHO53KSN were associated with trastuzumab resistance in HER-2-positive BC patients [42]. Compared with sensitive individuals, these two tRFs were upregulated in trastuzumab-resistant patients. The higher the expression level of tRF-30-JZOYJE22RR33 and tRF-27-ZDXPHO53KSN in HER-2-positive BC patients, the shorter the progression-free survival of the patient.

These functional tRFs with easy-to-detect properties may be used as potential biomarkers and intervention targets in clinical treatment. However, as a specific biomarker, it is still necessary to clarify the specificity and applicability of these tRFs among different types of cancer. As an efficient intervention target, the role and mechanism of these tRFs in cancer still needs to be fully explored.

## Conclusion and future perspectives

Although the recognition of the existence of tRNA breakdown products as cancer markers has been noted since the 1970s [84], the exact function of tRFs in cancer has only recently been the subject of direct study. The application of high-throughput sequencing technology has led to the discovery of the aberrant expression of many tRFs in a variety of cancer types. Some of these tRFs have been collated and deposited in several databases, which in turn has facilitated further discovery and functional research relating to tRFs. tRFs are constitutively generated in human cells, in both normal and stress conditions, and their composition and abundances are shaped by many factors, including gender, population origin, tissue, and disease subtype [36]. Increasing evidence suggests that tRFs are involved in many aspects of cancer and can be used as potential non-invasive biomarkers for the diagnosis and prognosis of cancer.

According to our review, most of these functional tRFs can regulate gene expression and/or translation and affect cellular signaling cascades in order to regulate the proliferation, metastasis, and drug resistance of tumor cells. However, the mechanisms of how tRFs regulate carcinogenesis and progression are largely unknown. Meanwhile, the depth and breadth of the biological function of tRFs seem significantly different in different types of cancer. Considering the abnormal expression of many tRFs in most cancer types, we believe that these tRFs may also play important functions in these cancer types. Further functional research will help to identify tRFs as diagnostic and prognostic biomarkers and therapeutic targets for clinical application.

We also realize that the expression of tRFs varied greatly with different types of cancer, but the underlying mechanism for the differential expression of tRFs is still poorly understood. The discovery of SHOT-RNAs in breast and prostate cancer led to the suggestion that the status of the hormone receptors may impact the production of tRFs in hormone-dependent tumors [66]. In addition, hypoxia-induced tRFs play important roles in BC [32, 63] and other stress-induced tiRNAs are also found to be important to breast and colon cancer [65, 94]. Interestingly, a recent study showed that in several instances, the abundance of tRFs is regulated without changes in mature tRNA levels. This suggests that tRNA genes can be selectively expressed to regulate noncanonical tRNA functions performed by tRFs without compromising the mature tRNA pool [39]. The modification status of tRNA nucleotides can affect stress-induced endonuclease activities and have an important impact on the tRNA fragmentation process [95]. For example, the m1A demethylated tRNA was found to be more sensitive to ANG cleavage that generates tiRNAs [96]. Another recent study has demonstrated that the 2′-O-methylation at position 34 of human elongator tRNA^Met^(CAT) prohibits site-specific cleavage of tRNA^Met^ (CAT) into tRFs by the stress-responsive endoribonuclease ANG [97]. All of these results reveal the complexity and challenge in the determination of exactly what conditions can cause tRF production, and whether this production is related to epigenetic modifications of tRNAs and/or to certain characteristics of cancer progression, and consequently how these tRNA/tRF modifications contribute to tumorigenesis and progression.

## Data Availability

All data generated or analyzed during this study are included in this published article.

## Abbreviations

tRNAs: Transfer RNAs
tRFs: tRNA fragments
mRNAs: Messenger RNAs
ncRNAs: Non-coding RNAs
lncRNAs: Long non-coding RNAs
sncRNAs: Small non-coding RNAs
tiRNAs: tRNA-derived stress-induced RNAs
ANG: Angiogenin
PAR-CLIP: Photoactivatable-Ribonucleoside-Enhanced Cross*l*inking and Immuno*p*recipitation
AGO: Argonautes
RNAi: RNA interference
miRNAs: MicroRNAs
siRNAs: Small interfering RNAs
SG: Stress granules
RG4: RNA G-quadruplex
RPS28: Ribosomal protein S28
TEs: Transposable elements
LTR: Long terminal repeat
ERV: Endogenous retrovirus
dsRNAs: Double-stranded RNAs
BC: Breast cancers
TNBC: Triple-negative breast cancer
NCL: Nucleolin
FZD3: Frizzled class receptor 3
SHOT-RNAs: Sex hormone-dependent tRNA-derived RNAs
RUNX1: Runt-related transcription factor 1
PRAD: Prostate adenocarcinoma
TCGA: The Cancer Genome Atlas
AR: Androgen receptor
CLL: Chronic lymphocytic leukemia
HTLV-1: Human T cell leukemia virus type 1
ATLL: Adult T cell leukemia/lymphoma
RPA1: Replication protein A1
NSCLC: Non-small cell lung cancer
AURKA: Aurora kinase A
CRC: Colorectal cancer
HCC: Hepatocellular carcinomas
EVs: Extracellular vesicles
AUC: Area under the curve
RPM: Reads per million
ROC: Receiver operating characteristic
